# The Association between Primary Tooth Emergence and Anthropometric Measures in Young Adults: Findings from a Large Prospective Cohort Study

**DOI:** 10.1371/journal.pone.0096355

**Published:** 2014-05-13

**Authors:** Ghazaleh Fatemifar, David M. Evans, Jonathan H. Tobias

**Affiliations:** 1 Medical Research Council Integrative Epidemiology Unit, School of Social and Community Medicine, University of Bristol, Bristol, United Kingdom; 2 Diamantina Institute, Translational Research Institute, University of Queensland, Brisbane, Queensland, Australia; 3 Musculoskeletal Research Unit, School of Clinical Sciences, University of Bristol, Bristol, United Kingdom; Université de Poitiers, France

## Abstract

Cross sectional studies suggest a link may exist between tooth emergence and obesity. To explore this relationship, we aimed to evaluate the prospective associations between primary tooth emergence and anthropometric measures in young adults. Multivariable linear regression was used to analyse relationships between primary tooth emergence, and anthropometric measures measured at 17.8 years, in 2977 participants (1362 males and 1615 females) from the Avon Longitudinal Study of Parents and Children (ALSPAC). In minimally adjusted models, ‘quintiles of number of paired teeth’ (assessed by questionnaire at 15 months) was positively associated with height [change in height (cm) per quintile increase in ‘number of paired teeth’ (β) = 0.35 (95%CI: 0.18, 0.52) P = 0.0001] and weight [ratio of geometric mean weight per quintile increase in ‘number of paired teeth’ (RGM) = 1.015 (95%CI: 1.010, 1.019) P<0.0001]. The relationship with weight was largely driven by fat mass, which showed an equivalent relationship with ‘quintiles of number of paired teeth’ to that seen for weight [RGM = 1.036 (95%CI: 1.022, 1.051) P<0.0001] (adjusted for height)]. Conversely, no association was seen between ‘quintiles of number of paired teeth’ and lean mass. An increase in ‘quintiles of number of paired teeth’ at age 15 months was associated with a higher Tanner stage at age 13 in girls but not boys, but further adjustment of associations between ‘quintiles of number of paired teeth’ and anthropometric traits for Tanner stage was without effect. Primary tooth emergence is associated with subsequent fat mass, suggesting these could share common constitutive factors, and that early primary tooth emergence may represent a hitherto unrecognised risk factor for the development of obesity in later life.

## Introduction

Thirty percent of children in Western countries such as the UK are overweight or obese [1], which has major adverse implications for public health [2], [3]. For example, paediatric obesity increases the risk of cardiovascular risk factors such as hypertension and insulin resistance, impacting on life expectancy [2]–[4]. Previous investigations have attempted to identify key environmental, genetic and constitutive factors which contribute to the risk of childhood obesity, in order to understand the pathological processes involved, and enable strategies to identify those at risk in whom preventative therapies should be targeted. For example, our recent genome wide association study (GWAS) of tooth emergence identified associations with variants in genes that have been implicated in obesity, *HMGA2* and *BMP4*, suggesting a hitherto unrecognised link may exist between tooth emergence and obesity [5], [6]. For example, a signalling pathway involving BMP4 and its receptor BMPR1A influences insulin secretion and may contribute to obesity [7]–[9]; mice lacking the *HMGA2* homolog had a reduction in fat mass and were resistant to diet induced obesity [10].

A small number of observational studies have previously examined associations between tooth emergence and obesity, primarily from a standpoint of obesity as a determinant of permanent tooth emergence. For example, Hilgers *et al* found that BMI was positively associated with dental age, in 104 children aged 8–15 [11]. Results were confirmed in a larger study of 5434 participants aged between 5 and 14 years, with obese children having on average 1.44 more teeth [12]. Similarly, in 110 Mexican children, children in higher BMI categories had greater number of teeth than children in lower categories [13]. Taken together, these studies suggest that there is an association between obesity and tooth emergence, but due to their cross sectional nature, it is not possible to establish cause and effect.

Here, we aimed to establish whether a common pathway is likely to exist between primary tooth emergence and adiposity, by performing a longitudinal analysis based on the Avon Longitudinal Study of Parents and Children (ALSPAC). To establish whether tooth emergence in early life is related to adiposity in early adulthood, we initially examined whether the former is related to weight as assessed at age 17. Tooth emergence may act as marker for growth and/or maturation, and so associations with body weight may not reflect differences in fat mass per se. Furthermore, any association with fat mass may be secondary to altered trajectories of growth and/or maturation. Therefore, further analyses were performed to establish whether (i) equivalent associations to those between tooth emergence in early life and weight at age 17 are seen for fat mass, (ii) associations between tooth emergence and fat mass are explained by other anthropometric differences such as those in height and/or lean mass, and (iii) relationships between tooth emergence and fat mass are explained by rate of maturation, using information about stage of puberty as assessed at age 13 to provide an estimate of age of puberty onset.

## Materials and Methods

### Participants

ALSPAC is a geographically based UK cohort that recruited pregnant women residing in Avon (South-west England) with an expected date of delivery between April 1^st^ 1991 and December 31^st^ 1992. A total of 15,247 pregnancies were enrolled with 14,775 children born (see www.alspac.bris.ac.uk for more information) [14]. Of these births, 14,701 children were alive at 12 months. Both mothers and children have been extensively followed from the 8th gestational week onwards using a combination of self-reported questionnaires, medical records and physical examinations. Ethical approval was obtained from the ALSPAC Law and Ethics committee and relevant local ethics committees, and written informed consent provided by all parents. The present study is based on questionnaires to the mother when children were a mean age of 15.3 months [SE = 0.02; CI: (15.26–15.34)], and a research clinic to which the whole cohort was invited, held when children were a mean age of 17.8 years, which was attended by 5084 participants.

### Dentition phenotypes

Tooth emergence phenotypes of the children were derived from questionnaires completed by the mothers and included questions regarding the ‘age at first tooth’ (assessed at 15months) and the ‘number of teeth’ in the child's mouth (at 15 months). ‘Age at first tooth’ was reported to the nearest month. The ALSPAC study website contains details of all the data that is available through a fully searchable data dictionary (http://www.bristol.ac.uk/alspac/researchers-/data-access/data-dictionary/).

### Outcomes and other co-variables

In those attending the age 17.8 research clinic, height was measured using a Harpenden stadiometer (Holtain Ltd., Crymych, UK), and weight was estimated to the nearest 50 g using a Tanita Body Fat Analyzer (model TBF 305). Measures of total body fat mass and lean mass were obtained from whole body DXA scans performed on a Lunar Prodigy. Birth weight was extracted from hospital records. Gestational age was calculated from the last menstrual period (from medical records) and the actual date of delivery. Tanner stage data was applied when participants were 13 years of age, based on pubic hair development as ascertained from a self-completion Tanner stage questionnaire. Mothers were asked to record the occupation for both herself and her partner, the highest of which was used to group participants into a head of household social class (classes I–V, with III split into manual and non-manual). This was created using the Office of Population Censuses and Surveys (1991).

### Statistical methods

Descriptive data was reported as means, standard deviations, medians and inter-quartile range (IQR). In order to analyse the relationships between ‘number of teeth’ and anthropometric measures, we categorised participants into five approximately equal sized groups (using xtile in stata) based on number of paired of teeth (teeth are known to erupt in pairs), both for clarity and to maximize statistical power. Multivariable linear regression was used to examine the relationship between independent variables; pairs of teeth quintiles and ‘age at first tooth’ and dependent variables height, weight, fat mass and lean mass, using an Ordinary Least Squares approach (OLS). Due to the skewed nature of weight and fat mass a natural log transformation was applied for analyses and back transformed so regression coefficients represent the ratio of geometric means (RGM) of the outcome variable per unit increase in the exposure variable. A ratio of geometric means has the same meaning as that of a conventional means; it is just that the estimate of the mean is more accurate as it takes account of the skewed nature of the distribution. In our base model, we adjusted for age at completion, age at DXA and gestational age to account for any variation of these measurements to the results of our analyses. Fat and lean mass were further adjusted for height alone, and height + fat/lean mass. Further models were explored with additional adjustment for Tanner stage. Analyses were stratified by sex and compared by including an interaction term for sex and primary tooth variables. All analyses were conducted using stata version 12.

## Results

### Characteristics of Participants

2977 participants (1362 males, 1615 females) were identified with complete data for primary tooth variables in early life and anthropometric variables at age 17. Males had slightly more teeth than females at 15 months, and age at emergence of first tooth was slightly earlier (Table 1). At age 17.8 years, boys were taller than girls and had greater lean mass, whereas fat mass was higher in girls. Overall, 21% of the study population were overweight or obese (defined as BMI>25); in comparison, the health survey for England in 2011 reported that approximately 30% of children in the UK aged between 2–15 were overweight or obese [15].

**Table 1:** Descriptive Table.

	Sex	Mean	(SD)	Median	P25	P75
**EARLY LIFE VARIABLES**						
**Number of Paired Teeth (15 months)**	Males	4.9	1.58	5	4	6
	Females	4.8	1.66	5	4	4
**Age at First Tooth (Assessed at 15 months)**	Males	6.9	2.3	7	5	8
	Females	7.2	2.3	7	5	9
**Gestational Age (Weeks)**	Males	39.3	1.9	40	39	41
	Females	39.6	1.6	40	39	41
**ANTHROPEMETRIC VARIABLES**						
**Height (cms)**	Males	178.9	6.7	178.8	174.5	183.1
	Females	165.5	6.2	165.2	161.3	169.5
**Weight (kg)**	Males	72.3	13.6	69.9	63.1	78.3
	Females	62.5	11.9	60.7	54.3	67.8
**Fat Mass (kg)**	Males	13.9	10.3	10.8	6.8	17.3
	Females	21.3	9.2	19.4	15.0	25.2
**Lean Mass (kg)**	Males	55.1	6.3	54.9	50.8	59.0
	Females	38.0	4.2	37.8	35.2	40.6

Descriptive statistics of ALSPAC participants with data for primary tooth variables, gestational age, and anthropometric variables as assessed at mean age 17.8 years. N males  = 1362, N females  = 1615. Number of paired teeth were categorised into 5 approximately equal sized groups (1 = 21.6%, 2 = 24.1%, 3 = 18.2%, 4 = 23.9%, 5 = 12.2%).

### Associations between Primary Tooth Variables and Anthropometric Variables

Initially we explored the relationship of primary tooth emergence with height and weight at age 17 (Table 2). ‘Quintiles of number of paired teeth’ was positively associated with height at age 17, particularly in boys (males: β = 0.58; CI 0.31, 0.84; females: β = 0.16; CI −0.06, 0.39; P = 0.01 for gender interaction). Conversely, ‘age of first tooth’ was inversely related to height at age 17, although in this instance relationships in boys and girls were broadly equivalent (males: β = −0.28; CI −0.44, −0.12; females: β = −0.16; CI −0.29, −0.03; P = 0.2 for gender interaction). ‘Quintiles of number of paired teeth’ was positively associated with weight at 17, to a similar extent in both sexes (males: RGM = 1.017; CI 1.010, 1.024; females: RGM = 1.013; CI 1.006, 1.019), whilst age at first tooth was inversely associated with weight (males: RGM = 0.994; CI 0.990, 0.998; females: RGM = 0.995; CI 0.992, 0.999).

**Table 2:** Relationship between Tooth Emergence and Height and Weight.

Number of Paired Teeth		Height							Weight						
	**Sex**	**N**	**β**	**95% CI**		**P**			**N**	**RGM**	**95% CI**		**P**		
	**Male**	1362	0.58	0.31	0.84	<0.0001			1362	1.017	1.010	1.024	<0.0001		
	**Female**	1615	0.16	−0.06	0.39	0.1572	**p(int)**	**R^2^**	1615	1.013	1.006	1.019	0.0001	**p(int)**	**R^2^**
	**Both**	2977	0.35	0.18	0.52	0.0001	0.01	0.52	2977	1.015	1.010	1.019	<0.0001	0.17	0.16
**Age at First Tooth**															
	**Sex**	**N**	**β**	**95% CI**		**P**			**N**	**RGM**	**95% CI**		**P**		
	**Male**	1362	−0.28	−0.44	−0.12	0.0007			1362	0.994	0.990	0.998	0.0063		
	**Female**	1615	−0.16	−0.29	−0.03	0.0140	**p(int)**	**R^2^**	1615	0.995	0.992	0.999	0.0163	**p(int)**	**R^2^**
	**Both**	2977	−0.21	−0.31	−0.11	<0.0001	0.2	0.53	2977	0.995	0.992	0.998	0.0003	0.5	0.15

Relationship between primary tooth variables (quintiles of ‘number of paired teeth’ at age 15 months and ‘age at first tooth’) and height (cm)/weight (Kg) at 17 years of age, in 1362 males and 1615 females. In the case of height, β-coefficients represent mean change in height per quintile increase in number of paired teeth/month increase in age at first tooth. Due to the skewed nature of weight we used the natural logarithm in analyses. Beta-coefficients were back transformed so that the coefficients represent the ratio of geometric means (RGM) of weight per quintile increase in number of paired teeth/month increase in age at first tooth. Analyses were adjusted for age at dxa scan, age (in months) of dentition questionnaire completion, gestational age and stratified by gender. p(int) is the p-value from a gender interaction test. R^2^ reports the coefficient of determination (variance explained) by the model outlined above.

We then explored the relationship between primary tooth variables and weight, by examining whether this could be explained by the association with height, and by studying whether a specific body compartment was primarily responsible for this relationship. In minimally adjusted analyses of the relationship between primary tooth emergence and fat mass, ‘quintiles of number of paired teeth’ and ‘age at first tooth’ were respectively positively and inversely associated with fat mass, with little evidence of a gender interaction (Table 3, basic model). The relationship between ‘quintiles of number of paired teeth’ and fat mass showed little attenuation after successive adjustment for height and lean mass, a positive association still being observed in the fully adjusted model (RGM = 1.033; CI 1.018, 1.047). On the log scale it is the difference in the expected geometric mean of the log of fat mass (GM = 15.16). On the original scale the ratio of geometric mean represents the percentage difference in fat mass per quintile change in number of paired teeth (i.e. 3% increase in fat mass per quintile change in number of paired teeth). Conversely, the association between ‘age at first tooth’ and fat mass was largely attenuated by these adjustments (RGM = 0.995; CI 0.987, 1.003).

**Table 3:** Relationship between Tooth Emergence and Fat Mass.

Number of Paired Teeth	Basic Model								Adjusted for height						Adjusted for height + lean mass					
	**Sex**	**N**	**RGM**	**95% CI**		**P**	**)**		**RGM**	**95% CI**		**P**	**p(int)**		**RGM**	**95% CI**		**P**		
	**Male**	1362	1.055	1.028	1.083	0.0001			1.048	1.021	1.076	0.0004			1.046	1.019	1.073	0.0007		
	**Female**	1615	1.029	1.013	1.044	0.0002	**p(int**	**R^2^**	1.027	1.012	1.042	0.0004		R^2^	1.022	1.008	1.037	0.0021	**p(int)**	**R^2^**
	**Both**	2977	1.040	1.026	1.055	<0.0001	0.58	0.22	1.036	1.022	1.051	<0.0001	0.83	0.24	1.033	1.018	1.047	<0.0001	0.89	0.27
**Age at First Tooth**																				
	**Sex**	**N**	**RGM**	**95% CI**		**P**			**RGM**	**95% CI**		**P**			**RGM**	**95% CI**		**P**		
	**Male**	1362	0.988	0.973	1.003	0.1263			0.991	0.976	1.007	0.2646			0.993	0.978	1.008	0.3631		
	**Female**	1615	0.993	0.985	1.002	0.1067	**p(int**	**R^2^**	0.995	0.986	1.003	0.2214	**p(int**	**R^2^**	0.996	0.988	1.004	0.3280	**p(int**	**R^2^**
	**Both**	2977	0.991	0.983	0.999	0.0304	0.98	0.22	0.993	0.985	1.002	0.1080	0.85	0.24	0.995	0.987	1.003	0.2018	0.65	0.26

Relationship between primary tooth variables (quintiles of ‘number of paired teeth’ at age 15 months and ‘age at first tooth’) and fat mass (kg) at 17 years of age, in 1362 males and 1615 females. Due to the skewed nature of fat mass we used the natural logarithm in analyses. Beta-coefficients were back transformed so that the coefficients represent the ratio of geometric means (RGM) of fat mass per quintile increase in number of paired teeth/month increase in age at first tooth. The basic model was adjusted for age at dxa scan, age (in months) of dentition questionnaire completion, gestational age and stratified by gender. p(int) is the p-value from a gender interaction test. R^2^ reports the coefficient of determination (variance explained) by the models outlined above.

We then analysed the relationship between primary tooth emergence and lean mass. ‘Quintiles of number of paired teeth’ and ‘age of first tooth’ were respectively positively and inversely related to lean mass in minimally adjusted analyses (Table 4, basic model). The relationship between ‘quintiles of number of paired teeth’ and lean mass was attenuated by adjustment for height, as reflected by a decrease in beta coefficient of approximately 50%. This resulted in further attenuation after additional adjustment for fat mass, such that in the final model there was little evidence of an association (β = 0.09; CI −0.02, 0.21). Similarly, there was little evidence of an association between ‘age at first tooth’ and lean mass in the fully adjusted model (β = −0.05; CI −0.11, 0.02).

**Table 4:** Relationship between Tooth Emergence and Lean Mass.

Number of Paired Teeth	Basic Model								Adjusted for height						Adjusted for height + fat mass					
	**Sex**	**N**	**β**	**95% CI**		**P**			**β**	**95% CI**		**P**			**β**	**95% CI**		**P**		
	**Male**	1362	0.47	0.22	0.72	0.0002			0.14	−0.06	0.34	0.1699			0.10	−0.10	0.30	0.3290		
	**Female**	1615	0.19	0.04	0.35	0.0144	**p(int)**	**R^2^**	0.12	0.00	0.25	0.0457	**p(int)**	**R^2^**	0.06	−0.06	0.17	0.3336	**p(int)**	**R^2^**
	**Both**	2977	0.32	0.18	0.46	<0.0001	0.06	0.72	0.15	0.03	0.26	0.0107	0.68	0.83	0.09	−0.02	0.21	0.0933	0.7	0.84
**Age at First Tooth**																				
	**Sex**	**N**	**β**	**95% CI**		**P**			**β**	**95% CI**		**P**			**β**	**95% CI**		**P**		
	**Male**	1362	−0.25	−0.40	−0.10	0.0011			−0.09	−0.21	0.03	0.1345			−0.08	−0.20	0.04	0.1752		
	**Female**	1615	−0.10	−0.19	−0.01	0.0236	**p(int)**	**R^2^**	−0.03	−0.10	0.04	0.3366	**p(int)**	**R^2^**	−0.02	−0.08	0.05	0.6535	**p(int)**	**R^2^**
	**Both**	2977	−0.17	−0.25	−0.08	0.0001	0.09	0.73	−0.06	−0.13	0.00	0.0693	0.24	0.83	−0.05	−0.11	0.02	0.1481	0.21	0.84

Relationship between primary tooth variables (quintiles of ‘number of paired teeth’ at age 15 months and ‘age at first tooth’) and lean mass (kg) at 17 years of age, in 1362 males and 1615 females. β-coefficients represent mean change in lean mass per quintile increase in number of paired teeth/month increase in age at first tooth. The basic model was adjusted for age at dxa scan, age (in months) of dentition questionnaire completion, gestational age and stratified by gender. p(int) is the p-value from a gender interaction test. R^2^ reports the coefficient of determination (variance explained) by the models outlined above.

In further analyses, associations between primary tooth emergence and anthropometric variables which we observed were unaffected by additional adjustment for birth weight and head of household social class (Tables S1, S2 and S3).

### Puberty-adjusted Analyses

Like primary tooth emergence, puberty is related to both skeletal maturation and body composition. To explore whether the relationship between primary tooth emergence and fat mass shares a common pathway with puberty, we examined whether (i) primary tooth variables were related to onset of puberty, and (ii) whether the associations between tooth emergence and height and fat mass persist after puberty adjustment, in 977 males and 1240 females matched to Tanner stage data from age 13. ‘Quintiles of number of paired teeth’ at age 15 months was positively associated with Tanner stage at age 13 in girls but not boys [OR (i.e. odds of being in a higher pubertal category per quintile increase in number of paired teeth) in boys: OR: 1.03, CI: 0.95, 1.12, P = 0.4; girls: OR: 1.15; CI: 1.07, 1.24, P<0.00001 (P = 0.10 for gender interaction)]. ‘Age of first tooth’ was inversely associated with Tanner stage at age 13 in girls but not boys [OR (i.e. odds of being in a higher pubertal category per month increase in age of first tooth) in boys: OR: 1.01, CI: 0.96, 1.06, P = 0.7; girls: OR: 0.94; CI: 0.90, 0.98, P<0.01 (P = 0.06 for gender interaction)].

We then examined the impact of additional adjustment for Tanner stage on associations between primary tooth emergence and height and fat mass, in the subset with Tanner stage data from age 13. As observed in the larger dataset, ‘quintiles of number of paired teeth’ and ‘age at first tooth’ were respectively associated with positive and inverse associations with height, particularly in boys (Table 5, basic model). Likewise, ‘quintiles of number of paired teeth’ was positively associated with fat mass, to a similar extent in both sexes, whereas there was little evidence of an association between ‘age at first tooth’ and fat mass (Table 5, adjusted for height + lean mass). These associations were unaffected by further adjustment for Tanner stage.

**Table 5:** Relationship between Tooth Emergence and Height/Fat Mass Adjusted for Tanner Stage.

Versus Height		Basic model							Adjusted for Tanner Stage					
**a) Number of Paired Teeth**														
	**Sex**	**N**	**β**	**95% CI**		**P**			**β**	**95% CI**		**P**		
	**Male**	977	0.50	0.19	0.81	0.0017			0.50	0.19	0.81	0.0017		
	**Female**	1240	0.03	−0.23	0.29	0.8297	**p(int)**	**R^2^**	0.06	−0.20	0.32	0.6544	**p(int)**	**R^2^**
	**Both**	2217	0.24	0.04	0.44	0.0204	0.01	0.52	0.25	0.05	0.45	0.0150	0.02	0.52
**Age at First Tooth**														
	**Sex**	**N**	**β**	**95% CI**		**P**			**β**	**95% CI**		**P**		
	**Male**	977	−0.25	−0.44	−0.06	0.0095			−0.25	−0.44	−0.06	0.0095		
	**Female**	1240	−0.10	−0.24	0.05	0.2122	**p(int)**	**R^2^**	−0.11	−0.26	0.04	0.1575	**p(int)**	**R^2^**
	**Both**	2217	−0.16	−0.28	−0.04	0.0068	0.12	0.52	−0.17	−0.28	−0.05	0.0058	0.18	0.52

Versus Fat Mass			Adjusted for Height + Lean Mass						Adjusted for Height + Lean Mass + Tanner Stage					
**b) Number of Paired Teeth**														
	**Sex**	**N**	**RGM**	**95% CI**		**P**			**RGM**	**95% CI**		**P**		
	**Male**	977	1.050	1.019	1.081	0.0013			1.049	1.019	1.081	0.0015		
	**Female**	1240	1.026	1.009	1.043	0.0021	**p(int)**	**R^2^**	1.023	1.007	1.040	0.0059	**p(int)**	**R^2^**
	**Both**	2217	1.037	1.021	1.054	<0.0001	0.97	0.28	1.035	1.019	1.052	<0.0001	0.96	0.28
**Age at First Tooth**														
	**Sex**	**N**	**RGM**	**95% CI**		**P**			**RGM**	**95% CI**		**P**		
	**Male**	977	0.985	0.967	1.003	0.0980			0.985	0.967	1.002	0.0904		
	**Female**	1240	0.997	0.988	1.006	0.5075	**p(int)**	**R^2^**	0.998	0.989	1.007	0.6841	**p(int)**	**R^2^**
	**Both**	2217	0.992	0.983	1.002	0.1017	0.78	0.28	0.993	0.983	1.002	0.1241	0.69	0.28

Relationships between primary tooth variables (quintiles of ‘number of paired teeth’ at age 15 months and ‘age at first tooth’) and height (cm)/fat mass (kg) at 17 years of age, with or without adjustment for Tanner stage as ascertained at age 13, in 977 males and 1240 females. Analyses with fat mass were additionally adjusted for height and lean mass. In the case of height, β-coefficients represent mean change in height per quintile increase in number of paired teeth/month increase in age at first tooth. Due to the skewed nature of fat mass we used the natural logarithm in analyses. Beta-coefficients were back transformed so that the coefficients represent the ratio of geometric means (RGM) of fat mass per quintile increase in number of paired teeth/month increase in age at first tooth. The basic model was adjusted for age at dxa scan, age (in months) of dentition questionnaire completion, gestational age and stratified by gender. p(int) is the p-value from a gender interaction test. R^2^ reports the coefficient of determination (variance explained) by the models outlined above.

**Figure 1:**
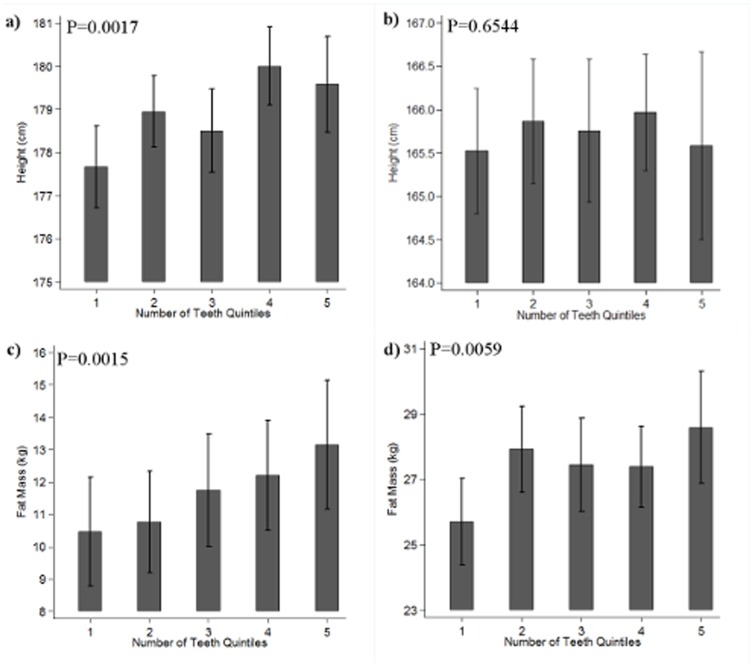
Relationship between ‘Number of Paired Teeth’ and Height/Fat-mass Adjusted for Tanner Stage and Stratified by Gender. Bar chart showing the relationship between quintiles of ‘number of paired teeth’ (15 months) and: a) height (cm) stratified for males and adjusted for tanner stage at 13 years, b) height (cm) stratified for females and adjusted for tanner stage at 13 years, c) Fat mass stratified for males and adjusted for height (cm), lean mass (kg) and tanner stage at 13 years. d) Fat mass stratified for females and adjusted for height (cm), lean mass (kg) and tanner stage at 13 years. All analyses were also adjusted for age of participant at dental questionnaire completion, age at dxa clinic, and gestational age. P-values report the P trend. N males  = 997, N females = 1240.

Finally, we explored the dose-response relationship between primary tooth variables and height and fat mass, by comparing means of height and fat mass across quintiles of ‘number of paired teeth’ in our final model incorporating Tanner stage. The association between ‘number of paired teeth’ and height showed a positive trend across quintiles in males, whereas no relationship was evident in females (Figure 1a and 1b respectively). Positive trends in fat mass were observed across quintiles for ‘number of paired teeth’, in both males and females (Figure 1c and 1d respectively).

## Discussion

In the present study, we examined relationships between primary tooth emergence and anthropometric traits at age 17. In our fully adjusted model, ‘quintiles of number of paired teeth’ and ‘age at first tooth’ were found to be positively and negatively related to height in males respectively, whereas no association was seen in females. Conversely, ‘quintiles of number of paired teeth’ was positively associated with fat mass in both males and females, whereas no relationship was seen with ‘age at first tooth’. The observed relationships were relatively strong. For example, a quintile increase in ‘number of paired teeth’ at 15 months was associated with 3.5% increase in (geometric) mean of fat mass at age 17.

To our knowledge, no previous investigation has examined relationships between primary tooth emergence and anthropometric parameters in later life. However, our findings are consistent with a small number of previous studies indicating positive associations between tooth emergence, height, and obesity in early childhood. For example, length at birth was positively associated with dental age (as measured by tooth emergence) over the first three years of life in 697 children [16]. In a sample of 359 children, length of birth was positively related to tooth emergence at 6 months [17]. Although we found the relationship between primary tooth emergence and height to be restricted to males, these previous studies revealed no indication of a gender difference. Gender interactions of this nature often fail to replicate, particularly in instances where it is difficult to understand the biological rationale. Nevertheless, the evidence for a gender interaction was relatively strong in statistical terms, suggesting that further studies are justified to explore the basis of these findings.

As discussed in the introduction, several previous cross sectional studies have reported positive associations between emergence of teeth and obesity in childhood, primarily from the standpoint of permanent dentition as the outcome [11]–[13]. These studies used BMI to evaluate obesity, rather than the more accurate DXA method employed in this study. BMI is thought to be a good indicator of excess body fat in more obese individuals, although differences in thinner participants largely reflect fat free mass [18].

While the present study differed in that we examined primary as opposed to permanent tooth emergence, the same measure, namely ‘number of teeth’ was evaluated, alongside the age at which deciduous teeth first emerge Although we identified associations between number of teeth and anthropometric measures at age 17, we observed little association between fat mass and ‘age at first tooth’. This is in spite of the fact that ‘number of teeth’ and ‘age at first tooth’ are correlated with each other relatively strongly r^2^ = 0.37 (p<0.00001). In terms of possible explanation for this apparent discrepancy, ‘age at first tooth’ involves historical data at the time the questionnaire is completed, in contrast to ‘number of teeth’. Hence, the former measure may be more imprecise, increasing the possibility of a type II error.

The process of tooth emergence is complex and highly regulated. Prior to emergence, mononuclear cells move to the dental follicle and fuse to produce osteoclasts, which then resorb alveolar bone to form an eruption pathway [19]. Studies in mice have implicated several signalling pathways as being critical for tooth emergence and development including those involving the gene families *BMP*, *EDA*, *FGF*, *SHH* and *WNT* [20]–[22]. Given the relationship with height discussed above, presumably, primary tooth emergence is also influenced by general growth regulatory processes. Consistent with this interpretation, GWAS studies have identified associations between primary tooth emergence and several growth-related genes e.g. *HMGA2*, *IGF2BP3*, *C6orf173* and *RAD51L1* [5], [6]. Furthermore, in monogenic disorders exemplified by Prader-Willie syndrome (caused by the deletion or inactivation of genes on the paternal strand in the region of 15q11-13), delayed tooth emergence occurs in association with growth and developmental delay [23], [24].

Therefore, in exploring the relationship between primary tooth emergence and fat mass, it is important to consider the role of growth and developmental factors as possible mediators. In particular, since our measure of total body fat mass is strongly influenced by height and lean mass, the relationship between ‘quintiles of number of paired teeth’ and fat mass might have been explained by greater body size. Since the association persisted after adjustment for height and lean mass as measured at age 17, this did not appear to be explained by ultimate body size, but does not preclude a role of accelerated growth and development in earlier life.

In terms of possible maturational factors, early puberty onset is associated with an increased risk of subsequent obesity [25]. Furthermore, we found evidence that earlier primary tooth emergence is associated with earlier onset of puberty in girls. The latter observation was contrary to a previous study based on 50 French-Canadian girls, which found no association between tooth emergence and puberty [26]. Nonetheless, the possibility exists that primary tooth emergence influences adiposity and thus potentially risk of obesity via a pathway-involving rate of maturation. However, against this suggestion, the association between ‘quintiles of number of paired teeth’ and fat mass which we observed appeared to be unaffected by adjustment for Tanner stage.

To the extent that our findings represent a causal relationship between primary tooth emergence and subsequent fat mass, they raise the possibility that early tooth emergence may represent a hitherto unrecognised risk factor for obesity in later life. This understanding may be helpful in identifying children in whom public health measures to combat obesity need to be targeted. Our findings also raise the possibility that understanding the processes, which regulate tooth emergence, may help in identifying new causes of obesity. For example, our recent GWAS of primary tooth emergence identified variants within *BMP4* and *HMGA2* to be associated with ‘age of first tooth’ and ‘quintiles of number of paired teeth’ at one year [5]. These genes have also been associated with the development of obesity. Pathway analysis based on the top 20 hits suggested a role of a several signalling mechanisms in primary tooth emergence [5], which could conceivably also contribute to the development of obesity.

### Limitations

Due to the observational nature of the study, it is not possible to exclude confounding as an explanation for the associations, which we observed. Although possible confounding by socio-economic status was examined, other factors remain to be evaluated, such as nutritional status of the mother, in light of a previously reported association between maternal caloric supplementation during pregnancy and tooth emergence in the infant [27]. Furthermore, the 2977 participants on which this study was based represent a minority of the overall ALSPAC cohort, and may differ in important ways from the remainder, thereby limiting the generalisability of our findings.

## Conclusions

We examined relationships between primary tooth emergence and anthropometric traits in early adulthood. ‘Quintiles of number of paired teeth’ at age 15 months was positively related both to height (males only) and fat mass at age 17. The association of ‘quintiles of number of paired teeth’ with fat mass persisted after adjustment for height and lean mass, and associations with height and fat mass were both unaffected by adjustment for puberty onset. These findings suggest that primary tooth emergence and obesity could share common constitutive factors; raising the possibility that early primary tooth emergence might represent a hitherto unrecognised risk factor for the development of obesity in later life.

## Supporting Information

Table S1 **Association between ‘Number of Paired Teeth’ and Height in a Further Adjustment for Birth Weight and Head of Household Social Class.** Relationship between quintiles of ‘number of paired teeth’ at 15 months and height (cm) at age 17. β-coefficients represent mean change in height per quintile increase in ‘number of paired teeth’. Basic model was adjusted for age at dxa scan, age (in months) of dentition questionnaire completion, gestational age and sex. The second model was adjusted for the basic model and birth weight/head of household social class.
(DOCX)

Table S2 **Association between ‘Number of Paired Teeth’ and Weight in a Further Adjustment for Birth Weight and Head of Household Social Class.** Relationship between quintiles of ‘number of paired teeth’ at 15 months and weight (kg) at age 17. Due to the skewed nature of weight we used the natural logarithm in analyses. Beta-coefficients were back transformed so that the coefficients represent the ratio of geometric means (RGM) of weight per quintile increase in ‘number of paired teeth’. The basic model was adjusted for age at dxa scan, age (in months) of dentition questionnaire completion, gestational age and sex. The second model was adjusted for the basic model and birth weight/head of household social class.
(DOCX)

Table S3 **Association between ‘Number of Paired Teeth’ and Fat Mass in a Further Adjustment for Birth Weight and Head of Household Social Class.** Relationship between quintiles of ‘number of teeth paired’ at 15 months and fat mass (kg) at age 17. Due to the skewed nature of fat mass we used the natural logarithm in analyses. Beta-coefficients were back transformed so that the coefficients represent the ratio of geometric means (RGM) of fat mass per quintile increase in ‘number of paired teeth’. The basic model was adjusted for age at dxa scan, age (in months) of dentition questionnaire completion, gestational age, sex, height and lean mass. The second model was adjusted for the basic model and birth weight/head of household social class.
(DOCX)
