# Supplementary material for: The Association between Primary Tooth Emergence and Anthropometric Measures in Young Adults: Findings from a Large Prospective Cohort Study
Source: PLoS One. 2014 May 13;9(5):e96355. doi: 10.1371/journal.pone.0096355 (PMC4019483; doi:10.1371/journal.pone.0096355)
Supplement: Table S1 — Association between ‘Number of Paired Teeth’ and Height in a Further Adjustment for Birth Weight and Head of Household Social Class. Relationship between quintiles of ‘number of paired teeth’ at 15 months and height (cm) at age 17. β-coefficients represent mean change in height per quintile increase in ‘number of paired teeth’. Basic model was adjusted for age at dxa scan, age (in months) of dentition questionnaire completion, gestational age and sex. The second model was adjusted for the basic model and birth weight/head of household social class. (DOCX) [file pone.0096355.s001.docx]

**Table S1: Association between 'Number of Paired Teeth ' and Height in a Further Adjustment for Birth Weight and Head of Household Social Class**

|  |  | **Height** | | | | **Height + Birth Weight** | | | |
| --- | --- | --- | --- | --- | --- | --- | --- | --- | --- |
|  | **N** | **β** | **95% CI** | | **P** | **β** | **95% CI** | | **p** |
| **Number of Paired Teeth** | 2941 | 0.34 | 0.16 | 0.51 | 0.001 | 0.20 | 0.010 | 0.35 | 0.03 |
|  |  | **Height** | | | | **Height + HHSC** | | | |
|  | **N** | **β** | **95% CI** | | **P** | **β** | **95% CI** | | **p** |
| **Number of Paired Teeth** | 2864 | 0.33 | 0.15 | 0.51 | 0.0001 | 0.34 | 0.170 | 0.52 | <0.0001 |
|  |  |  |  |  |  |  |  |  |  |
