# Supplementary material for: The Association between Primary Tooth Emergence and Anthropometric Measures in Young Adults: Findings from a Large Prospective Cohort Study
Source: PLoS One. 2014 May 13;9(5):e96355. doi: 10.1371/journal.pone.0096355 (PMC4019483; doi:10.1371/journal.pone.0096355)
Supplement: Table S3 — Association between ‘Number of Paired Teeth’ and Fat Mass in a Further Adjustment for Birth Weight and Head of Household Social Class. Relationship between quintiles of ‘number of teeth paired’ at 15 months and fat mass (kg) at age 17. Due to the skewed nature of fat mass we used the natural logarithm in analyses. Beta-coefficients were back transformed so that the coefficients represent the ratio of geometric means (RGM) of fat mass per quintile increase in ‘number of paired teeth’. The basic model was adjusted for age at dxa scan, age (in months) of dentition questionnaire completion, gestational age, sex, height and lean mass. The second model was adjusted for the basic model and birth weight/head of household social class. (DOCX) [file pone.0096355.s003.docx]

**Table S3: Association between 'Number of Paired Teeth' and Fat Mass in a Further Adjustment for Birth Weight and Head of Household Social Class**

|  |  | **Fat Mass** | | | | **Fat Mass + Birth Weight** | | | |
| --- | --- | --- | --- | --- | --- | --- | --- | --- | --- |
|  | **N** | **RGM** | **95% CI** | | **p** | **RGM** | **95% CI** | | **p** |
| **Number of Paired Teeth** | 2941 | 1.03 | 1.02 | 1.05 | <0.0001 | 1.03 | 1.02 | 1.04 | <0.0001 |
|  |  | **Fat Mass** | | | | **Fat Mass + HHSC** | | | |
|  | **N** | **RGM** | **95% CI** | | **p** | **RGM** | **95% CI** | | **p** |
| **Number of Paired Teeth** | 2864 | 1.03 | 1.01 | 1.05 | <0.0001 | 1.03 | 1.02 | 1.05 | <0.0001 |
